# Estimating Kinetic Rate Parameters for Enzymatic Degradation of Lyophilized Silk Fibroin Sponges

**DOI:** 10.3389/fbioe.2021.664306

**Published:** 2021-07-06

**Authors:** Julie F. Jameson, Marisa O. Pacheco, Jason E. Butler, Whitney L. Stoppel

**Affiliations:** Department of Chemical Engineering, Herbert Wertheim College of Engineering, University of Florida, Gainesville, FL, United States

**Keywords:** silk fibroin, kinetic model, degradation, biomaterial, enzymes, lyophilized sponge

## Abstract

Sponge-like biomaterials formed from silk fibroin are promising as degradable materials in clinical applications due to their controllable breakdown into simple amino acids or small peptides *in vivo*. Silk fibroin, isolated from *Bombyx mori* silkworm cocoons, can be used to form sponge-like materials with a variety of tunable parameters including the elastic modulus, porosity and pore size, and level of nanocrystalline domains. These parameters can be independently tuned during formulation resulting in a wide parameter space and set of final materials. Determining the mechanism and rate constants for biomaterial degradation of these tunable silk materials would allow scientists to evaluate and predict the biomaterial performance for the large array of tissue engineering applications and patient ailments a priori. We first measured *in vitro* degradation rates of silk sponges using common protein-degrading enzymes such as Proteinase K and Protease XIV. The concentration of the enzyme in solution was varied (1, 0.1, 0.01 U/mL) along with one silk sponge formulation parameter: the level of crystallinity within the sponge. Additionally, two experimental degradation methods were evaluated, termed continuous and discrete degradation methods. Silk concentration, polymer chain length and scaffold pore size were held constant during experimentation and kinetic parameter estimation. Experimentally, we observed that the enzyme itself, enzyme concentration within the bulk solution, and the sponge fabrication water annealing time were the major experimental parameters dictating silk sponge degradation in our experimental design. We fit the experimental data to two models, a Michaelis-Menten kinetic model and a modified first order kinetic model. Weighted, non-linear least squares analysis was used to determine the parameters from the data sets and Monte-Carlo simulations were utilized to obtain estimates of the error. We found that modified first order reaction kinetics fit the time-dependent degradation of lyophilized silk sponges and we obtained first order-like rate constants. These results represent the first investigations into determining kinetic parameters to predict lyophilized silk sponge degradation rates and can be a tool for future mathematical representations of silk biomaterial degradation.

## Graphical Abstract

In this manuscript, we determine kinetic rate constants to describe the process of enzymatic degradation of lyophilized silk fibroin scaffolds. We present the experimental data and kinetic model used to arrive at these rate constants. Ultimately, we conclude that the determined rate constants vary as a function of the enzyme itself, the enzyme concentration, and the crystalline content within the scaffolds.

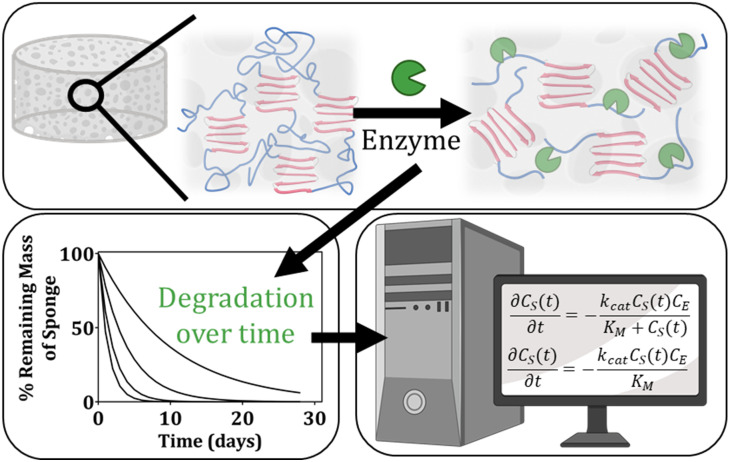

## Introduction

Biodegradable synthetic (Agrawal and Ray, 2001; Tyler et al., 2016; Chen et al., 2019) and natural (Aamodt and Grainger, 2016; Stoppel et al., 2017; Huang et al., 2018; Park and Woo, 2018; Wang et al., 2019) biomaterials have been utilized as scaffolds for tissue engineering and as platforms for *in vitro* culture to aid in tissue reconstruction and fundamental studies of developmental biology and disease pathology. For *in vivo* applications, knowledge of degradation rates is essential as the implanted materials should have controllable and predictable break down that complements the rate of neo-tissue formation and regeneration, providing necessary or complementary signaling modulators for wound healing and tissue repair (Tabata, 2001; Shin et al., 2003). To complicate these strategies, patients heal and remodel at different rates depending upon their age, hormone levels, and comorbidities (e.g., heart disease, diabetes). Currently, materials available to physicians for tissue reconstruction are not adaptable to the various injury environments or the remodeling rates of patients, rendering current commercially available surgical materials sub-optimal for many patients. Moreover, engineers and scientists are left to trial-and-error biomaterial design unless quantitative information is available on how various fabrication parameters impact biomaterial degradation rates *in vivo*. Elucidating the mechanisms behind degradation of biomaterials is critical to create novel scaffolds for specific patients, emphasizing the various rates of soft tissue remodeling that span the patient population.

Natural protein-based biomaterials are being researched since they degrade via proteolytic enzymes naturally found in the body and break down into small peptides or amino acids which are readily absorbed by surrounding metabolically active cells. Biomaterials from silk fibroin represent one class of natural protein-based biomaterials with demonstrated clinical potential (Vepari and Kaplan, 2007; Kundu et al., 2013, 2014; Stoppel et al., 2015a; Farokhi et al., 2018; Gholipourmalekabadi et al., 2020). For example, some regenerated silk products (Sofregen Medical, Inc., United States FDA 510K, Number K172545) are already FDA approved. While synthetic materials can have advanced properties (Wade et al., 2015; Austin and Rosales, 2019), translation to the clinic and FDA approval are slow. One major advantage of materials formed from silk fibroin is the slow enzymatic degradation and proteolysis of the material into simple amino acids or small peptides due to the nanocrystalline domains (Li et al., 2003; Brown et al., 2015; Rnjak-Kovacina et al., 2015; Kluge et al., 2016). Previous work has experimentally explored and tracked *in vitro* and *in vivo* degradation of silk fibroin platforms such as particles (K. Numata et al., 2010), fibers (Arai et al., 2004), films (Arai et al., 2004; Lu et al., 2011; Shang et al., 2013; Brown et al., 2015), hydrogels (Brown et al., 2015), sheets (Li et al., 2003), sponges (Wang et al., 2008; Rnjak-Kovacina et al., 2015), and yarns (Horan et al., 2005) by commonly used proteolytic enzymes (e.g., Collagenase, Protease XIV, a-Chymotrypsin, Proteinase K, etc.). The results from prior work demonstrate that the final structure and format of the silk material plays a major role in the rate of degradation (Guo et al., 2020). However, these experimental investigations have not related biomaterial formulation to rates of enzymatic degradation or developed mathematical representations of biomaterial breakdown and/or release of key biologics or therapeutics. This is desired and advantageous for on-going efforts in the development and optimization of silk biomaterials within the tissue engineering and regenerative medicine community.

Our lab and others have explored the use of silk fibroin-based sponges for *in vivo* applications (Rnjak-Kovacina et al., 2015; Stoppel et al., 2015b; Rameshbabu et al., 2018, 2020). To generate sponge-like structures that are water insoluble, the scaffolds are formed by freezing, lyophilizing, and autoclaving or water annealing regenerated *Bombyx mori* silk fibroin solution. For the purposes of this paper, they will be referred to as lyophilized silk sponges. An advantage of lyophilized silk sponges is that the scaffold structure is collapsible, meaning that when hydrated, one can squeeze the scaffold to remove all liquid, forming a thin, filter paper-like structure. Then, upon placing that same material back into liquid, the structure recovers to its original volume. Lyophilized silk sponges can also be tuned during formulation by modulating the silk polymer chain length, the concentration of silk protein incorporated into the sponge, the temperature at which the silk solution is frozen (dictating sponge pore size), and the level of induced crystallinity (β-sheet content) within the silk proteins (Wray et al., 2012; Mandal et al., 2013; Rnjak-Kovacina et al., 2014, 2015; Stoppel et al., 2015b). These structures have substantial potential for *in vivo* use for applications in regenerative engineering and drug delivery, but optimizing the scaffold architecture and initial properties is currently performed by trial and error.

Herein, we describe measurements of the *in vitro* enzymatic degradation of lyophilized silk sponges and conduct kinetic modeling with the aim of determining accurate, mathematical representations of the degradation rates. Experimentally, degradation rates of silk materials were measured *in vitro* using common protein-degrading enzymes such as Proteinase K and Protease XIV. The concentration of the enzyme in solution was varied (1, 0.1, 0.01 U/mL) along with one silk sponge formulation parameter: the level of crystallinity within the samples (2–12 h of water annealing). Enzymes were chosen to match previously published data with silk fibroin biomaterials (Guo et al., 2020). In addition, the cleavage sites of these two enzymes are well understood and documented, providing the knowledge to understand the underlying mechanisms behind silk fibroin degradation. The enzyme concentrations were chosen to capture the degradation of the silk sponges over time periods that allowed for adequate data collection for kinetic modeling. However, it is important to note that Protease XIV is a non-mammalian enzyme cocktail (Catalog No. P5147, Sigma Aldrich, St. Louis, MO) and these concentrations are not based on *in vivo* data. Thus, more work will be required for translation to *in vivo* studies. Additionally, two methods of performing the degradation studies were explored: continuous and discrete. We held the silk concentration, polymer chain length and scaffold pore size constant as an initial set of experimental data for model development and validation. Two kinetic models were explored (Michaelis-Menten kinetic model and modified first order kinetic model), and reaction rate constants were determined from fitting the experimental data. The results provide quantitative information on the rates of degradation over a range of factors such as enzyme concentration, enzyme type, and water annealing time.

## Experimental Methods

### Silk Fibroin Extraction

Silk fibroin solution was prepared as previously described through isolation from the cocoons of *Bombyx mori* silkworms (Rockwood et al., 2011). Briefly, pure silk fibroin protein was isolated by degumming 5 grams of silk cocoons in 2 L of boiling 0.02 M sodium carbonate (Catalog No. 451614, Sigma Aldrich, St. Louis, MO) for 30 min. Once degumming was complete, the fibroin protein was air-dried in the fume hood for over 48 h and solubilized by denaturation in 9.3 M aqueous lithium bromide (Catalog No. 213225, Sigma Aldrich, St. Louis, MO) at 60°C for 4 h. Then, the solution was dialyzed with a 3.5 kilodalton (kDa) molecular weight cutoff dialysis membrane tubing (Spectrum^TM^ Spectra/Por^TM^ 3 RC Dialysis Membrane Tubing, 3,500 Dalton MWCO, Catalog No. 08-670-5C, Thermo Fisher Scientific, Waltham, MA) to remove the lithium bromide ions in deionized water. The solubilized silk solution was centrifuged twice (>2,000 g, 20 min, 4°C) to remove insoluble particles. The concentration of the silk solution was determined by drying a known volume of the silk solution at 60°C and massing the remaining solids. This protocol resulted in a 5–7% weight per volume (wt./v, 0.05–0.07 g/mL) silk solution. Silk solutions were stored at 4°C for a maximum of 3 weeks prior to use in making silk sponges.

### Silk Sponge Fabrication

Following published protocols (Rnjak-Kovacina et al., 2015), isotropic silk sponges were formed by pouring 4 mL of silk solution (3% wt./v, 0.03 g/mL) into wells of a 12 well plate and freezing the solution in −80°C freezer overnight. Following freezing, silk sponges were lyophilized at −80°C and 0.185 mbar for 5–7 days (FreeZone 12 Liter −84°C Console Freeze Dryer, Labconco, Kansas City, MO). The sponge-like scaffolds were made to be water insoluble by water annealing (WA) at room temperature under −0.05 MPa vacuum pressure with 500 mL of ultrapure Milli-Q water for 0, 2, 6, or 12 h and samples are referred to as 2, 6, and 12 h samples as a function of the WA time. The vacuum desiccator (Bel-Art SP^TM^ Scienceware^TM^ Lab Companion Round Style Vacuum Desiccator, Catalog No. 08-648-100, Thermo Fisher Scientific, Waltham, MA) has a volume of 6L. This induces β-sheet formation in the silk fibroin, resulting in silk protein crystallization (Jin et al., 2005; Hu et al., 2011). For continuous degradation experiments, scaffolds were cut using a 3 mm radius biopsy punch to create a cylinder 4 mm in height. For discrete degradation experiments, scaffolds were cut using a 2 mm diameter biopsy punch with a plunger to create a cylinder 2 mm in height (Supplementary Figure 1A).

### Fourier Transform Infrared Spectroscopy (FTIR) Analysis

FTIR analysis was executed to determine the secondary structure of silk sponges following water annealing for 0, 2, 6, or 12 h. Silk sponges were analyzed via a Nicolet iS50 FTIR Spectrometer (Thermo Fisher Scientific, Waltham, MA) using an attenuated total reflection (ATR) germanium crystal. The spectrometer was used with an MCT/A detector and KBr beamsplitter. To obtain absorbance spectra, the samples were subjected to 128 scans at scanning resolution of 4 cm^–1^ over the wavenumber range of 4,000–650 cm^–1^. For each sample, background spectra were collected under the same conditions and subtracted from each scan. The amide I region (1,590–1,710 cm^–1^) can be split into regions based on the secondary structure: 1,605–1,615 cm^–1^ as side chain/aggregated strands, 1,616–1,637 cm^–1^ and 1,697–1,703 cm^–1^ as β-sheet structure, 1,638–1,655 cm^–1^ as random coils, 1,656–1,662 cm^–1^ as α-helical bands, and 1,663–1,696 cm^–1^ as turns (Xiao Hu et al., 2006).

### Enzymatic Degradation of Silk Sponges

#### Enzymatic Degradation of Silk Sponges (3 mm Radius by 4 mm Height)—Continuous Method

To experimentally determine the rate of silk sponge degradation *in vitro* over time, silk sponges were immersed in enzyme solution over time and the mass of the sponge was determined at specific time points. To begin, the number of samples required for a biological replicate of *n* = 3 for each time point (Days 1–7, 11, 14, 21, and 28), enzyme (Proteinase K, Protease XIV), enzyme concentration (1 and 0.1 U/mL), and water annealing time (6 and 12 h) was calculated. To reduce the number of samples, the time points were divided into three sections: Sponge A (Days 1, 4, 7, and 21), Sponge B (Days 2, 5, 11, and 28), and Sponge C (Days 3, 6, and 14). The mass of dry silk sponges was recorded, and the silk sponges were placed in 1.5 mL Eppendorf tubes with 1 mL of Proteinase K (1 and 0.1 U/mL) in PBS, Protease XIV (1 and 0.1 U/mL) in PBS, or PBS solution alone. Proteinase K (Lot # 118M4089V, Catalog No. P6556, Sigma Aldrich, St. Louis, MO) had an activity of 30 U/mg. Protease XIV (Lot # SLCB5967, Catalog No. P5147, Sigma Aldrich, St. Louis, MO) had an activity of 6.4 U/mg. The Eppendorf tubes were incubated with continuous shaking at 37°C and the enzyme solution or PBS was removed and replaced every 48 h. For a given time point, the silk belonging to the section was massed while the other silk sponges remained in enzyme solution and were not handled. Prior to weighing, liquid was removed by first squeezing the sponge followed by aspiration of any remaining enzyme solution. On days 1–7, 11, 14, 21, and 28, the mass of the silk sponge was recorded. Silk sponge degradation was calculated as % remaining mass compared to original silk sponge mass. This method is described and depicted in Supplementary Figure 1B and Supplementary Table 1.

#### Enzymatic Degradation of Silk Sponges (1 mm Radius by 2 mm Height)—Discrete Method

To avoid potential errors associated with periodic sampling of silk sponges in the continuous method, experiments were also conducted in which the sponge was sampled and then discarded. For these discrete measurements, the silk sponges were of a different size (1 mm radius by 2 mm height). During this experiment, empty 1.5 mL Eppendorf tube masses were recorded. Additionally, the dry silk sponges were placed in the 1.5 mL Eppendorf tubes of known mass and then the mass of the 1.5 mL Eppendorf tube and silk sponge was recorded. Next, 0.5 mL of Proteinase K (0.01 and 0.1 U/mL) in PBS was added to the 1.5 mL Eppendorf tube with silk sponge. Proteinase K (Lot # 118M4089V, Catalog No. P6556, Sigma Aldrich, St. Louis, MO) had an activity of 30 U/mg. The Eppendorf tubes were incubated with continuous shaking at 37°C and the enzyme solution was removed and replaced every 48 h. For a given time point, a subset of samples (*n* = 5) was removed from incubation and the enzyme solution was aspirated out of the 1.5 mL Eppendorf tube. The Eppendorf tubes with the silk sponges were dried at 60°C in the oven for 2 h. The 1.5 mL Eppendorf tube and dry silk sponge mass was recorded. Silk sponge degradation was calculated as % remaining mass compared to original silk sponge mass. This method is described and depicted in Supplementary Figure 1C and Supplementary Table 2.

### Statistical Methods

Experimental degradation data are expressed as mean ± standard deviation (SD). GraphPad Prism 8.4.1 (La Jolla, CA) was utilized to analyze these data. Degradation data was analyzed using multiple *t*-tests where consistent standard deviations was not assumed. Statistical significance is reported as *p* ≤ 0.05. Rate constant parameters were determined from weighted least squares analysis and the standard deviation was calculated from Monte Carlo studies.

## Theoretical Basis

Given that the degradation of lyophilized silk scaffolds has not been previously modeled, we evaluated the applicability of two different kinetic models using our experimental data: Michaelis-Menten kinetics and first-order reaction kinetics. We used data collected from silk sponge samples stored under rocking conditions at 37°C, as described in section “Enzymatic Degradation of Silk Sponges”. Results from estimating parameters in the two kinetic models, as given in section “Kinetic Modeling and Parameter Estimation to Describe Lyophilized Silk Scaffold Degradation,” allow us to more clearly differentiate the effects of the different conditions used in the experiments. Additionally, the analysis suggests that one of the models is a better approximation to the experimental observations than the other. Here we briefly describe both models.

We assume that the enzymatic degradation is the dominant kinetic mode for the breakdown of the silk sponges, while recognizing that the physical properties of the scaffold likely influence the degradation. Accordingly, we describe silk scaffold breakdown using a traditional enzymatic pathway,

(1) $$E+S\,\begin{array}{c} k_{1}\\ ⇌\\ k_{-1} \end{array}\,E\,S\overset{k_{c\,a\,t}}{⟶}E+P.$$

In this reaction pathway, *E* represents the enzyme, *S* is the silk sponge substrate, *ES* is the enzyme-silk sponge substrate complex, and *P* represents the peptides of silk fibroin protein that are liberated from the substrate due to the enzymatic reaction. The rate constants *k_1*, *k*_*–1*_, and *k*_*cat*_ correspond to the forward, reverse, and catalytic rate constants, respectively.

Assuming that the concentration of the enzyme-substrate complex (*C*_*ES*_(*t*)) is constant,

(2) $$\frac{\partial C_{E\,S}\,\left(t\right)}{\partial t}=0,$$

gives the Michaelis-Menten kinetic model (Seibert and Tracy, 2014). This model predicts that the rate of silk sponge substrate disappearance is given by

(3) $$\frac{\partial C_{S}\,\left(t\right)}{\partial t}=\frac{-k_{c\,a\,t}\,C_{S}\,\left(t\right)\,C_{E}}{K_{M}+C_{S}\,\left(t\right)},$$

where the Michaelis-Menten constant is defined as

(4) $$K_{M}=\frac{k_{-1}+k_{c\,a\,t}}{k_{1}}.$$

The concentration of enzymes is *C_E* and the time dependent concentration of the silk sponge substrate material is *C*_*S*_(*t*). The concentration is calculated using

(5) $$C_{S}\,\left(t\right)=\frac{M_{S}\,\left(t\right)}{\pi \,R^{2}\,h},$$

where *M*_*S*_(*t*) is the experimentally measured mass of the substrate at time *t*. The conversion to a concentration occurs by normalizing all obtained silk substrate masses with the initial volume of the scaffold which is cylindrical and has a radius *R* and height *h*. Note that the generation of silk peptides is equal and opposite to the rate of silk sponge substrate disappearance,

(6) $$\frac{\partial C_{P}\,\left(t\right)}{\partial t}=\frac{-\partial C_{S}\,\left(t\right)}{\partial t},$$

where *C*_*P*_(*t*) is the time dependent concentration of silk peptides. The substrate concentration can be determined by numerically solving Equation (3) with the correct initial concentration.

Michaelis-Menten kinetics have been widely used to model enzymatic reactions (Cheng and Prud’homme, 2000; Fennouri et al., 2013; Andersen et al., 2018). Previously, silk fibroin degradation by elastase was modeled using Michaelis-Menten kinetics by Hu and colleagues (Hu et al., 2008). They found that degradation by elastase followed Michaelis-Menten kinetics with a *K_M* of 14.49 mg/mL.

Despite previous successes in describing the degradation of silk-based material with the Michaelis-Menten model, we also considered a modified first-order reaction. This model, which is first order with respect to *C*_*S*_(*t*) and *C_E*, is given by

(7) $$\frac{\partial C_{S}\,\left(t\right)}{\partial t}=\frac{-k_{c\,a\,t}}{K_{M}}\,C_{S}\,\left(t\right)\,C_{E}=-k_{f}\,C_{S}\,\left(t\right)\,C_{E},$$

and can be recovered from the Michaelis-Menten model if the substrate concentration is much smaller than *K_M*. The modified first order rate constant is defined as *k_f*. The substrate concentration can be determined analytically as a function of time:

(8) $$C_{S}\,\left(t\right)=C_{S\,0}\,e^{\left(-k_{f}\,C_{E}\,t\right)},$$

where *C*_*S0*_ is the initial concentration, *C*_*S*_(*t*=0)=*C*_*S*0_.

We emphasize that these models assume that the enzyme concentration is temporally and spatially uniform throughout the volume of the sponge and surrounding fluid. Likewise, the time-dependent concentration for the substrate is uniform throughout the sponge. These assumptions are consistent with the design of the experiments, where the samples were under constant agitation and fresh enzyme solution was supplied every 48 h to maintain constant activity. Also, diffusion is assumed to be unimportant, as the timescale for the degradation process is on the order of days.

## Results and Discussion

In regenerative medicine and tissue engineering, the goal is to develop materials for use in the body that aid in functional restoration of tissue. Achieving functional recovery requires a material that balances the regeneration and regrowth of tissue with the scaffold degradation. To strike this balance, biomaterials scientists must identify which properties of the material and the material formulation drive degradation. Additionally, development of a kinetic model and evaluation of the associated rate constants can aid in quantifying the dependence of the degradation on parameters such as enzyme type and concentration. Consequently, here we characterize the enzymatic degradation of the material and impact of formulation parameters on the reaction model and rate constants.

First in section “Kinetic Modeling and Parameter Estimation to Describe Lyophilized Silk Scaffold Degradation,” we explore the use of modified first order reaction kinetics vs. Michaelis-Menten reaction kinetics to describe experimental results of lyophilized silk scaffold degradation. The results of the kinetic modeling are presented and discussed in the subsequent sections which examine the effects of the enzyme type, enzyme concentration, water annealing time, and more.

### Kinetic Modeling and Parameter Estimation to Describe Lyophilized Silk Scaffold Degradation

**FIGURE 1:**
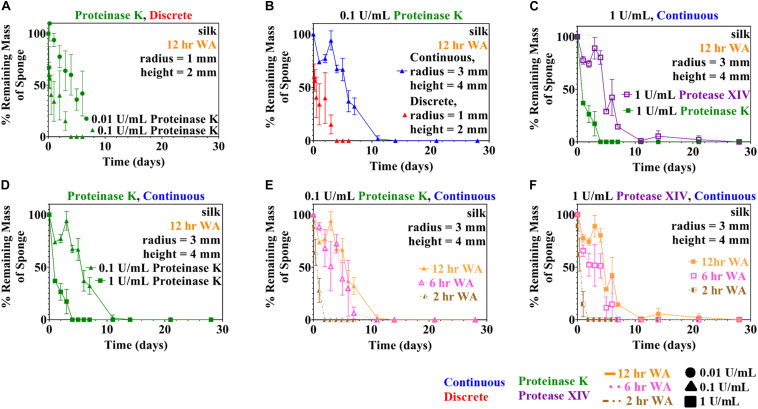
Experimental quantitative changes of silk sponge mass during enzymatic degradation using two methods (discrete and continuous) are presented here. **(A)** Silk sponges were immersed in 0.01 and 0.1 U/mL using the discrete method to find the change in mass over time. Parameter estimation of modified first order rate constants was carried out using the data obtained in **(A)**. Silk sponges were immersed in Proteinase K at a concentration of 0.1 U/mL at 37°C and both experiment types were carried out: **(B)** continuous method and discrete method. **(C)** Using the continuous method, silk sponges were subjected to incubation with Protease XIV and Proteinase K at 1 U/mL and 37°C. Proteinase K (1 U/mL) increased the rate of degradation over Protease XIV (1 U/mL). **(D)** Silk sponges subjected to Proteinase K at 0.1 and 1 U/mL using the continuous method demonstrate silk degradation is dependent on the concentration of the enzyme. Sponges water annealed (WA) for 2, 6, and 12 h were immersed in **(E)** Proteinase K (0.1 U/mL) and **(F)** Protease XIV (1 U/mL). Degradation of 6 h WA annealed silk sponges was only slightly faster than degradation of 12 h WA silk sponges for both **(E)** Proteinase K (0.1 U/mL) and **(F)** Protease XIV (1 U/mL). The ordinate on all graphs is expressed as the mass of the sample divided by the starting point mass multiplied by 100. Data expressed by mean ± 1SD. Continuous method had biological replicates of *n*=3 and the discrete method had biological replicates of *n*=5.

Both rate laws described in section “Theoretical Basis” were examined: the Michaelis-Menten model (Equation 3) and the modified first order reaction model (Equation 7). Analysis initially was performed using data from the discrete experiments using 12 h WA silk sponges immersed in 0.01 and 0.1 U/mL Proteinase K (see Figure 1A), since there was more data available for this set of conditions. Analysis of other conditions make use of conclusions from this set of data.

Multiple methods of estimating parameters for Michaelis-Menten rate laws have been proposed (Atkins and Nimmo, 1975; Toulias and Kitsos, 2016). We used a Newton-Raphson method to find the parameter values *k*_*cat*_ and *K_M* that minimize the weighted square error between the data and the model,

(9) $$E=\sum_{i}^{N_{s}}\left[\left(w_{i}\right)*{\left(C_{S}\,\left(t_{i}\right)-C_{m}\,\left(t_{i}\right)\right)}^{2}\right]$$

where *C*_*S*_(*t*_*i*_) and *w_i* are the measured concentration and inverse variance of the measurement at time *t_i*. The number of time measurements for each set of conditions is *N_s*, and the mean and variance at each time are calculated from all data collected at that time. Note that variances, and hence weights, are generally different at each time *t_i*. The model predictions for the substrate concentration at each corresponding time, *C*_*m*_(*t*_*i*_), were generated numerically. To find the parameters, the value of *k*_*cat*_ that minimizes *E* was found over a large range of *K_M* values; then, the values of *k*_*cat*_ and *K_M* at which *E* is minimum were identified. This procedure avoided the inherent instability of seeking both parameters simultaneously, while still being relatively fast to evaluate.

**FIGURE 2:**
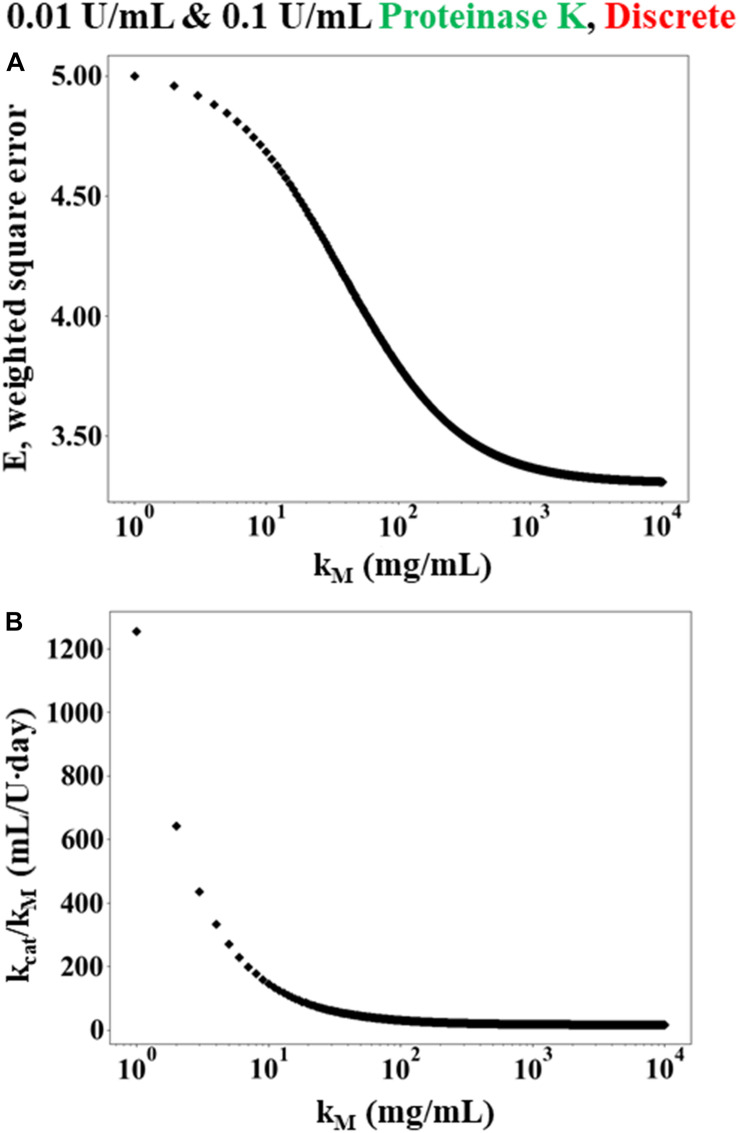
Simulated results of weighted least squares analysis using the Michaelis-Menten rate law for data obtained from discrete experiments using 12 h WA silk sponges immersed in 0.01 and 0.1 U/mL Proteinase K. **(A)** The minimum error *E* plotted as a function *K_M*. The value of *E* decreases monotonically as *K_M* increases. **(B)** The corresponding value of $\frac{k_{c\,a\,t}}{K_{M}}$ which minimizes *E* at each value of *K_M*.

Figure 2 shows that the error *E* decreases as *K*_*M*_→∞ while the ratio of *k*_*cat*_/*K*_*M*_ asymptotes to a constant value. Since *K*_*M*_→∞, the rate law given in Equation (3) can be rewritten as Equation (7). Consequently, Equation (8) was used in place of the numerical solution of *C*_*m*_(*t*_*i*_) within the weighted least squares Equation (9). Minimizing the weighted least squares using a Newton-Raphson method gave a value of 15 mL/U⋅day for *k_f*. Using the enzyme activity (30 U/mL) and molecular weight (28,900 g/mol), *k_f* can be expressed equivalently as 0.0052 mL/mg⋅s and 150.5 L/mol⋅s.

To determine the error in the estimate of *k_f*, a Monte-Carlo study was performed. Multiple sets of data (10,000) were generated randomly using the mean values and standard deviations of the measured data while assuming a Gaussian distribution for the errors. This simulated data was then processed using least squares analysis, with a resulting estimate for the mean and standard deviation for the modified first order reaction rate of *k_f* = 15 mL/U⋅day with a standard deviation of 3 mL/U⋅day.

Monte-Carlo simulations were also performed on the Michaelis-Menten rate law to further assess the relative validity of the modified first order model. In this case, 1,000 simulated data sets were generated randomly and then the square error was minimized for each set using the procedure described above. The vast majority of the randomly generated data sets (>95%) resulted in a prediction of modified first order kinetics (i.e., the square error was minimized while *K*_*M*_→∞ and the ratio of *k*_*cat*_/*K*_*M*_ asymptoted to a constant value), similar to the case shown in Figure 2. The remaining samples predicted finite values of *K_M* ≈ 14 ± 36 mg/mL, which is similar to the value of 14.49 mg/mL that was found by Hu and colleagues (Hu et al., 2008) for silk fibroin degradation by elastase. Still, the overwhelming number of random trials indicating that *K*_*M*_→∞ strongly suggests that the modified first order model is more appropriate than the Michaelis-Menten model, even when accounting for the errors in the measured data.

The preceding analysis used the combined data for *C*_*E*_ = 0.01 and 0.1 U/mL to give *k_f* = 14 ± 3 mL/U⋅day. Repeating the analysis using each set of data independently returns *k_f* = 15 ± 3 and 13 ± 8 mL/U⋅day for *C*_*E*_ = 0.01 and 0.1 U/mL, respectively (see Table 1). Likewise, independently fitting the two data sets to the Michaelis-Menten model gives a minimum error as *K*_*M*_→∞, as did the analysis on the combined data sets. The consistency of the combined and independently determined parameters provides additional evidence that the first order model is more appropriate than the Michaelis-Menten model.

**TABLE 1:** Modified first order rate constant determined from weighted least squares analysis and standard deviation for k_*f*_ calculated using Monte Carlo studies for 12 h WA silk sponges immersed in varying concentrations of Proteinase K and Protease XIV.

Experiment type	Enzyme	Enzyme concentration (U/mL)	k_*f*_ (mL/U⋅day)
Discrete	Proteinase K	0.01 and 0.1	14 ± 3
Continuous	Proteinase K	0.1 and 1	1.0 ± 0.4
Discrete	Proteinase K	0.1	13 ± 8
Discrete	Proteinase K	0.01	15 ± 3
Continuous	Proteinase K	0.1	2.2 ± 0.7
Continuous	Proteinase K	1	1.0 ± 0.1
Continuous	Protease XIV	1	0.2 ± 0.03

*Water Annealing Time: 12 h.*

Hence, all the data sets were analyzed using the modified first order kinetic model. Table 1 shows the results for modified first order rate constant estimation and the accompanying standard deviation from Monte Carlo studies for 12 h WA silk sponges immersed in varying concentrations of Proteinase K and Protease XIV. The results given in the tables for the various conditions are discussed in the following sections. Also, we note that the substrate concentration predicted by the first-order model (Equation 7) can be re-written as

(10) $$\frac{M_{S}\,\left(t\right)}{M_{S}\left(t=0\right)}=e^{\left(-k_{f}\,C_{E}\,t\right)},$$

where the percent remaining mass of the sponge is $100*\frac{M_{S}\,\left(t\right)}{M_{S}\left(t=0\right)}$. Consequently, data regarding the degradation can be compared on this basis without needing to consider the initial substrate concentration or size of the initial sponge sample.

### Continuous vs. Discrete Sampling Methods in Accurate Depiction of Silk Scaffold Degradation

Two methods of data sampling, termed the “continuous method” and “discrete method,” were used following experimental methodologies reported in the literature (Shang et al., 2013; Gil et al., 2014; Brown et al., 2015; Malwela and Ray, 2015; Cardoso et al., 2016; Zhang et al., 2019; Guaresti et al., 2020). A comparison of degradation data collected using both methods is shown in Figure 1B for the case of 0.1 U/mL Proteinase K. The figure shows a difference in the rate of enzymatic degradation; for example, the continuous and discrete methods yield a normalized standard deviation at measurement point day 1 of 74 ± 2 and 34 ± 19%, respectively, for the degraded samples. This is further confirmed by the rate constants for these two conditions which vary by a factor of almost six (see Table 1).

These results indicate that the continuous and discrete sampling methods can give different results, though there are two possible explanations for the differences. The experiments shown in Figure 1B for the continuous method and the discrete method were performed at different times and with different batches of silk fibroin solution. Consequently, one possibility is that subtle variations in the synthesis of the sponges is causing the observed differences. The other strong possibility is that the differences are caused by the periodic sampling and handling of the lyophilized silk sponges required when using the “continuous” protocol.

We note that the continuous method was employed to obtain continuous release kinetics data and to maintain constant enzymatic activity throughout the experiment. The advantage of the continuous method is that it tracks the mass loss of a single sponge over time. The discrete method assumes constant enzymatic degradation over of the degradation timeline, eliminating issues with weighing and measuring the same sponge repeatedly. However, the discrete method cannot track a single silk sponge to completion. We continue to use data sets from both methods throughout the analyses presented here.

### Enzyme Specificity for the Substrate Impacts Silk Scaffold Degradation and Kinetic Rate Constant

To elucidate the role of enzyme composition on scaffold degradation, we first examined immersing sponges in 1 U/mL Protease XIV and 1 U/mL Proteinase K using the continuous method, as presented in Figure 1C. Utilizing the same concentration of each enzyme allowed us to elucidate the role of the number of enzyme cleavage sites in lyophilized silk sponges. The % remaining mass from sponges immersed in 1 U/mL Protease XIV became statistically similar to 0% mass or 0 mg at day 11 while sponges immersed in 1 U/mL Proteinase K became statistically similar to 0% mass or 0 mg at day 2 (Figure 1C and Table 2). Experimentally, we were able to determine that the enzyme itself (Proteinase K vs. Protease XIV) influences the rate of degradation. This agrees with previously published data (Li et al., 2003; Shang et al., 2013; Brown et al., 2015; Rnjak-Kovacina et al., 2015) and Table 3 addresses that cleavage sites and number of cleavage sites from the two enzymes differ for silk fibroin protein where the higher number of cleavage sites leads to a faster rate of degradation (Brown et al., 2015). Additionally, Proteinase K at 1 U/mL had a larger rate constant (*k*_*f*_=1.0 ± 0.1 mL/U⋅day) than Protease XIV at 1 U/mL (*k_f* = 0.2 ± 0.03 mL/U⋅day) which agrees with the data obtained from continuous degradation studies (Figure 1C and Table 1).

**TABLE 2:** The *t*-test analysis for each experiment with respect to 0% remaining sponge mass.

Experiment type	Enzyme	Enzyme concentration (U/mL)	Water annealing time	Day the mass is not statistically significant from 0% mass remaining	*P*-value of similarity to 0
Continuous	Proteinase K	0.1	12 h	day 11	0.374
Discrete	Proteinase K	0.1	12 h	N/A*	
Continuous	Proteinase XIV	1	12 h	day 11	0.201
Continuous	Proteinase K	1	12 h	day 3	0.61
Discrete	Proteinase K	0.01	12 h	N/A*	
Continuous	Proteinase K	0.1	6 h	day 6	0.121
Continuous	Protease XIV	1	6 h	day 5	0.126
Continuous	Proteinase K	0.1	2 h	day 2	0.341
Continuous	Protease XIV	1	2 h	day 2	0.341

*Experiment not run to completion.

**TABLE 3:** Proteolytic enzyme (Proteinase K, Protease XIV) information (Brown et al., 2015; Wongpinyochit et al., 2018; Guo et al., 2020).

Enzyme	Cleavage sites	# of Estimated cleavage sites on silk fibroin
Proteinase K	His, Phe, Trp, Tyr, Ala, Ile, Leu, Pro, Val, Met	∼2,200
Protease XIV	Tyr, Phe, Trp, His, Lys, Arg	∼390

Proteases such as Proteinase K and Protease XIV are serine proteases. They cleave peptide bonds adjacent to the aliphatic, aromatic, and hydrophobic amino acids. Proteinase K from *Engyodontim album* cleaves amide bonds adjacent to histidine, phenylalanine, tryptophan, tyrosine, alanine, isoleucine, leucine, proline, valine, and methionine (Table 3; Ebeling et al., 1974; Sweeney and Walker, 1993). While Protease XIV from *Streptomyces griseus* cleaves amide bonds adjacent to histidine, phenylalanine, tryptophan, tyrosine, lysine, and arginine (Table 3; Bauer, 1978).

**FIGURE 3:**
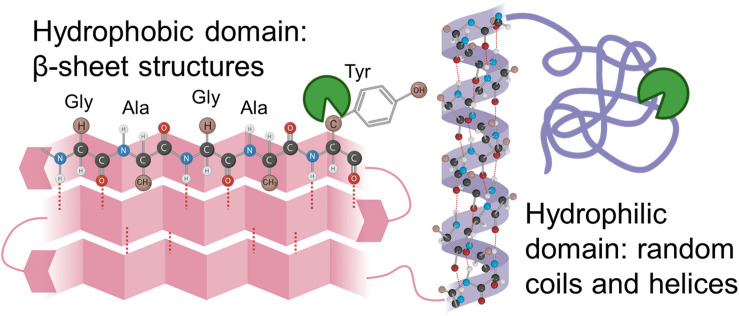
Schematic representation of silk fibroin with the hydrophobic domain (pink) and hydrophilic domain (purple) are demonstrated here. Hydrophilic domains are hypothesized to be cleaved first and allow enzyme solution into the hydrophobic domain. Inspiration and information came from Wang et al. (2019) and Guo et al. (2020).

Silk fibroin from *Bombyx mori* silk cocoons is made of a heavy chain, light chain, and P25 glycoprotein (Inoue et al., 2000; Zhou et al., 2001). The heavy chain of silk fibroin, the major constituent, will be focused on hereafter because the light chain and P25 glycoprotein account for only 5% of the amino acids present in silk fibroin from the *B. mori* (Inoue et al., 2000; Zhou et al., 2001). The silk fibroin heavy chain consists of 12 hydrophobic domains and 11 small hydrophilic domains (Figure 3). The hydrophobic domains contain β-sheet regions with repetitive amino acid motifs. The amino acid motifs consist of glycine-X (GX) repeats where X can be alanine, serine, or tyrosine (Figure 3). Brown and colleagues along with Wongpinyochit and colleagues did predictive calculations on the number of cleavage sites within the heavy chain of silk fibroin for many enzymes including Proteinase K and Protease XIV (Brown et al., 2015; Wongpinyochit et al., 2018). The predicted values for Proteinase K and Protease XIV were 2,200 and 390, respectively, given in Table 3. These values did not consider the accessibility of the cleavage sites due to secondary structure. However, the present data agrees where we find the greatest extent of degradation with 1 U/mL Proteinase K compared to 1 U/mL Protease XIV.

### Increased Enzyme Concentration Results in Faster Degradation of Silk Scaffolds

Silk sponges analyzed using the discrete (Figure 1A) or continuous (Figure 1D) method were degraded in different concentrations of the same enzyme to determine if enzyme concentration changes the rate of silk sponge degradation. With the continuous method, silk sponges were degraded in 0.1 and 1 U/mL Proteinase K at 37°C with gentle agitation. The % remaining mass of sponges immersed in 0.1 U/mL Proteinase K became statistically similar to 0% mass or 0 mg at day 11 while sponges immersed in 1 U/mL Proteinase K became statistically similar to 0% mass or 0 mg at day 2 (Figure 1D and Table 2). The rate constant, *k_f*, is 2.2 ± 0.7 mL/U⋅day for silk sponges in 0.1 U/mL Proteinase K and 1.0 ± 0.1 mL/U⋅day for silk sponges in 1 U/mL Proteinase K (Table 1).

With the discrete method, silk sponges were subjected to 0.01 and 0.1 U/mL Proteinase K at 37°C with gentle agitation. These two experiments were not run to completion, but the influence of enzyme concentration is still captured where 0.1 U/mL Proteinase K reduced the % mass more quickly than did 0.01 U/mL Proteinase K (Figure 1A and Table 2). The rate constant, *k_f*, is 14 ± 3 mL/U⋅day for silk sponges in 0.01 U/mL Proteinase K and 13 ± 8 mL/U⋅day for silk sponges in 0.1 U/mL Proteinase K (Table 1).

As expected, based on previous literature (Shang et al., 2013), the enzyme concentration influences silk sponge degradation. This demonstrates that the silk scaffold substrate concentration at these enzyme concentrations still have substrate available to bind the enzyme and thus increases the rate of degradation. However, there is a limit of maximum enzyme concentration where all the silk sponge substrate is bound, and the addition of more enzyme will not change the rate of degradation, but these concentration levels are not expected *in vivo*.

### Capturing Variation in β-Sheet Content in Kinetic Rate Parameters

**FIGURE 4:**
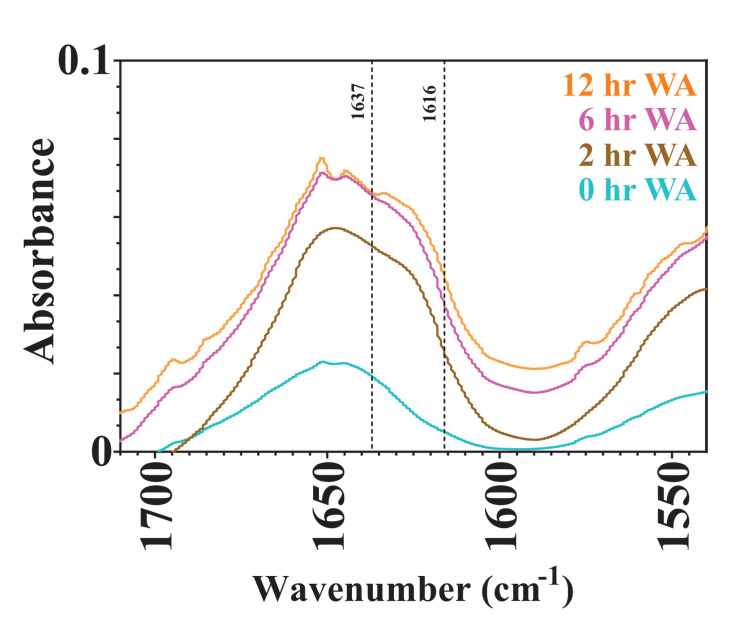
FTIR absorbance spectra of 0, 2, 6, and 12 h water annealed silk sponges. The dotted lines at 1,616 and 1,637 cm^– 1^ define the region of wavenumbers over which increased absorbance indicates the presence of β-sheet structures.

Varying the water annealing time was investigated to determine the influence of the β-sheet domains on substrate availability and the degradation rate. Fourier transform infrared spectroscopy (FTIR) results are shown in Figure 4 for silk sponges that were water annealed for 0, 2, 6, and 12 h. The 0 h WA silk sponge was analyzed as a negative control. The elevated absorbance between 1,616 and 1,637 cm^–1^ demonstrates that all samples other than the 0 h WA sponges contained β-sheet structures. Additionally, the 6 h WA silk sponge and the 12 h WA silk sponge have similar absorbance spectra and β-sheet content. The 2 h WA samples have a lower β-sheet content than the 6 and 12 h WA samples, as evidenced by the lower absorbance between 1,616 and 1,637 cm^–1^.

Using the continuous method, we measured enzymatic degradation of 2, 6, and 12 h WA silk sponges immersed in 0.1 U/mL Proteinase K (Figure 1E) and 1 U/mL Protease XIV (Figure 1F). As seen in Figure 1E and Table 2, the % mass remaining of 2 h WA sponges in 0.1 U/mL Proteinase K became statistically similar to 0% mass or 0 mg at day 2. The % mass remaining of 6 h WA sponges in 0.1 U/mL Proteinase K became statistically similar to 0% mass or 0 mg at day 6. This contrasts with the 12 h WA sponges in 0.1 U/mL Proteinase K where the % mass remaining became statistically similar to 0% mass or 0 mg at day 11. However, the large standard deviation on day 6 of the 6 h WA sponge could be skewing these results. It appears that the degradation of the 6 h WA silk sponges closely correspond to the degradation of the 12 h WA sponges (Figure 1E) with day 7 being the first day that they are vastly different from each other. The degradation of the 2 h WA sponges does not closely follow the degradation of the 6 h WA sponges or the 12 h WA sponges. By day 1, the 2 h WA sponges had already differentiated themselves from both the 6 h WA sponges and 12 h WA sponge’s degradation rates. Moreover, the rate constant, *k_f*, is 2.2 ± 0.7 mL/U⋅day for 12 h WA silk sponges (Table 1), 2.1 ± 0.8 mL/U⋅day for 6 h WA silk sponges (Table 4), and 11 ± 3.1 mL/U⋅day for 2 h WA silk sponges (Table 5).

**TABLE 4:** Modified first order rate constant determined from weighted least squares analysis for 6 h WA silk sponges immersed in Proteinase K and Protease XIV.

Experiment type	Enzyme	Enzyme concentration (U/mL)	k_*f*_ (mL/U⋅day)
Continuous	Proteinase K	0.1	2.1 ± 0.8
Continuous	Protease XIV	1	0.2 ± 0.08

*Water Annealing Time: 6 h.*

**TABLE 5:** Modified first order rate constant determined from weighted least squares analysis for 2 h WA silk sponges immersed in Proteinase K and Protease XIV.

Experiment type	Enzyme	Enzyme concentration (U/mL)	k_*f*_ (mL/U⋅day)
Continuous	Proteinase K	0.1	11 ± 3.1
Continuous	Protease XIV	1	1.4 ± 0.4

*Water Annealing Time: 2 h.*

Data in Figure 1F and Table 2 show that the % mass remaining of 2 h WA sponges in 1 U/mL Protease XIV became statistically similar to 0% mass or 0 mg at day 2. The % mass remaining of 6 h WA sponges became statistically similar to 0% mass or 0 mg at day 5 while the % remaining mass of 12 h WA sponges in 1 U/mL Protease XIV became statistically similar to 0% mass or 0 mg at day 11. Again, the 6 h WA silk sponges closely correspond to the degradation of the 12 h WA silk sponges while the 2 h WA sponges do not closely correspond to either the 6 h WA silk sponges or the 12 h WA silk sponges. The rate constant, *k_f*, is 0.2 ± 0.03 mL/U⋅day for 12 h WA silk sponges (Table 1), 0.2 ± 0.08 mL/U⋅day for 6 h WA silk sponges (Table 4), and 1.4 ± 0.4 mL/U⋅day for 2 h WA silk sponges (Table 5).

It was hypothesized that water annealing for shorter periods of time will increase the rate at which the silk sponge degrades because there will be fewer nanocrystalline domains and thus easier access for enzymes to cleave the silk fibroin protein. Rnjak-Kovacina et al. (2015) determined that lyophilized silk sponges that were autoclaved had a greater β-sheet content than lyophilized silk sponges that were untreated, or water annealed for 2 h. They further showed that the degradation of lyophilized silk sponges that had been autoclaved degraded slower than those that were water annealed for 2 h. Our results also show the dependence of water annealing time on the rate of degradation of silk sponges between the 2 h WA silk sponges and the 6 and 12 h WA silk sponges. The similar values of *k_f* for the 6 and 12 h WA silk sponges are due to the similar β-sheet content, as revealed by the similar FTIR spectra (Figure 4). Our results do suggest that the modified first order rate constant, *k_f*, can capture the difference in water annealing times in the silk sponge fabrication steps.

Enzymes are thought to degrade the amorphous, hydrophilic domains of the sponges (Figure 3) relatively rapidly (Numata et al., 2010; Park et al., 2020). The amorphous, hydrophilic domains contain the N-termini, C-termini, linker segments in the heavy chain, and the light chain. The crystalline domain of packed β-sheet structures degrades more slowly, as they break down due to hydrophobic collapse and interaction with water within the system (Park et al., 2020). Consequently, structural changes induced by processing the silk biomaterials with different water annealing times could be used to tune the silk scaffold degradation rates for use *in vivo*. However, current understanding through *in vitro* analysis does not necessarily coordinate to *in vivo* responses as the enzymes present at an implant site will be different from those explored within this manuscript. However, this *in vitro* work sets the stage for mathematically representing silk biopolymer degradation for future applications for predictable *in vivo* performance.

## Conclusion

Natural biomaterials have been leveraged for many applications both *in vitro* and *in vivo*, including as degradable implants for tissue repair and regeneration or as scaffolds for 3-dimensional tissue culture. However, for many biomaterial formulations, optimization of the starting material properties often leaves engineers and scientists using educated guess-and-check methods for determining optimal biomaterial formulations for a given application. Quantitative information regarding how various formulation parameters impact biomaterial degradation *in vivo* is difficult to obtain and often ethically questionable, as trying every material formulation *in vivo* is ineffective in terms of ethical justification of animal numbers as well as quite costly. Determining the mechanism and rate constants for biomaterial degradation of silk materials under various biological conditions would allow scientists to evaluate and predict the performance for the large array of tissue engineering applications and patient ailments a priori.

In this work, we aimed to find the parameters that dictate silk fibroin lyophilized sponge degradation rates and estimate the reaction rate constants to improve material optimization prior to *in vivo* experimentation. Degradation rates of silk materials were measured *in vitro* using common protein-degrading enzymes. We then evaluated two kinetic models and calculated reaction rate parameters from the experimental data. The degradation of lyophilized silk sponges fit a kinetic model described by modified first order reaction kinetics rather than Michaelis-Menten kinetics, and modified first order reaction parameters were tabulated for various enzymes, experimental methods, and silk sponge crystallinities. We observed that the enzyme, enzyme concentration, and water annealing time influence lyophilized silk sponge degradation *in vitro* when using enzymes, Proteinase K and Protease XIV. These results are the first step in developing a mathematical representation of silk scaffold degradation for guiding formulation of smart biomaterials that can meet the needs of clinicians and surgeons as they treat a wide array of patients and their ailments. Specifically, these kinetic parameters can be used for further validation with diffusion studies and presents the framework for investigating more clinically relevant enzymes, such as matrix metalloproteinases (MMPs). Thus, future work aims to explain which components of the original scaffold formulation more clearly (silk polymer chain length, concentration of silk protein incorporated into the sponge, temperature at which the silk solution is frozen) drive *in vivo* degradation.

## Data Availability Statement

The raw data supporting the conclusions of this article will be made available by the authors, without undue reservation.

## Author Contributions

WS and JJ conceived of the presented idea. JJ and JB developed the theory and performed the computations. JB verified the computations. WS oversaw experimental methods and design. JJ and MP executed the experiments. All authors discussed the results and contributed to the final manuscript.

## Conflict of Interest

The authors declare that the research was conducted in the absence of any commercial or financial relationships that could be construed as a potential conflict of interest.
