# Supplementary material for: Estimating Kinetic Rate Parameters for Enzymatic Degradation of Lyophilized Silk Fibroin Sponges
Source: Front Bioeng Biotechnol. 2021 Jul 6;9:664306. doi: 10.3389/fbioe.2021.664306 (PMC8290342; doi:10.3389/fbioe.2021.664306)
Supplement: Supplementary file 1 [file Data_Sheet_1.pdf]

Supplementary Material: Estimating kinetic rate parameters for enzymatic degradation of lyophilized silk fibroin sponges

Julie F Jameson[a], Marisa O Pacheco[a], Jason E Butler[a], and Whitney L Stoppel[a1]
[a]Department of Chemical Engineering, Herbert Wertheim College of Engineering, University of Florida, Gainesville, FL, USA 32611

[1]Correspondence:
Whitney L. Stoppel, PhD
1030 Center Drive
PO Box 116005
Gainesville, FL 32611
Whitney.Stoppel@ufl.edu

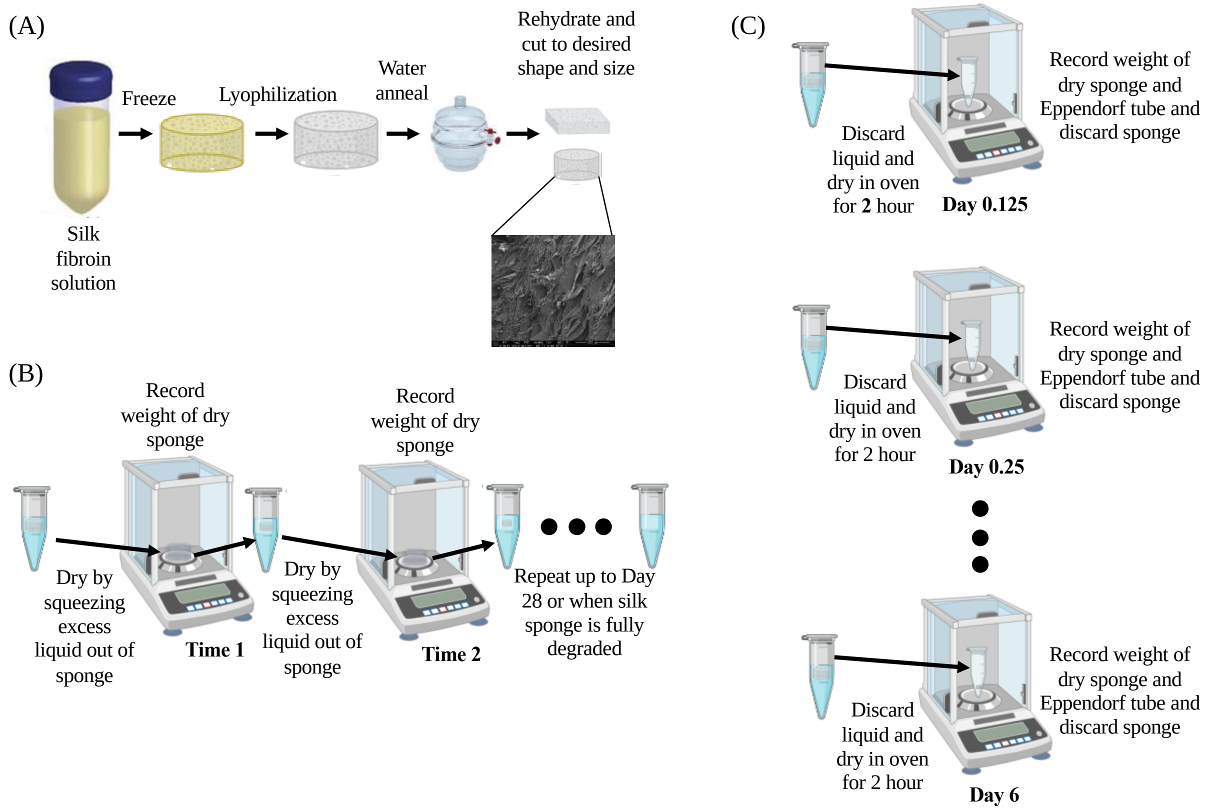

Supplementary Figure 1: (A) Presented is a schematic representation of silk sponge fabrication for degradation experiments. Aqueous silk solution is cast into wells of a 6-well plate. The 6-well plate is put in a -80ºC freezer overnight and then lyophilized at -80ºC and 0.185 mbar for 5-7 days. The silk sponges were then made to be water insoluble by water annealing at room temperature under -0.05 MPa vacuum pressure with 250 mL of ultrapure Mili-Q® water for 12 or 6 hours. After inducing β-sheet formation by water annealing, scaffolds were cut to the desired size. (B) The continuous method of sponge degradation is detailed here. Samples were divided into three sections named Sponge A, Sponge B, and Sponge C with a biological replicate of N=3. The specific time points for recording data for each sponge is detailed in Supplementary Table 1. The mass of dry silk sponges at the beginning of the experiment were recorded, and the silk sponges were placed in 1.5 mL Eppendorf tubes with 1 mL of enzyme solution or PBS. For a given time point, the silk sponge belonging to the section was dried by squeezing the silk sponge and the dry mass was recorded while the other silk sponges remained in enzyme solution and not handled. The silk sponge then was put back into the enzyme solution until the next time point or it was fully degraded. (C) The discrete method of sponge degradation use is detailed here. Silk sponges were placed in pre-weighed 1.5 mL Eppendorf tubes with 0.5 mL enzyme solution. At the time of sampling with an N=5, the enzyme solution was aspirated from the 1.5 mL Eppendorf tube to leave only the silk sponge in the 1.5 mL Eppendorf tube. The silk sponge in the 1.5 mL Eppendorf tube were weighed and the mass was recorded. The silk sponge was not used again. To find the mass lost over time, the recorded mass of the pre-weighed Eppendorf tube was subtracted from the recorded mass of the 1.5mL Eppendorf tube and dry silk sponge at the time of sampling. Supplementary Table 2 describes the timeline for this experiment.

Supplementary Table 1. Experimental timeline for the continuous method of degradation

	Time 1	Time 2	Time 3	Time 4
Sponge A	Day 1	Day 4	Day 7	Day 21
Sponge B	Day 2	Day 5	Day 11	Day 28
Sponge C	Day 3	Day 6	Day 14	

Radius of sponge: 3mm
Height of sponge: 4mm
Enzyme solution refreshed every 48 hours

Supplementary Table 2. Experimental timeline for the discrete method of degradation

	**Time 1**
Sponge A	Day 0.125
Sponge B	Day 0.25
Sponge C	Day 0.5
Sponge D	Day 1
Sponge E	Day 2
Sponge F	Day 3
Sponge G	Day 4
Sponge H	Day 5
Sponge I	Day 6

Radius of sponge: 1mm
Height of sponge: 2mm
Enzyme solution refreshed every 48 hours
